# Multiple resistance and influence of breeding sites on pyrethroid resistance in *Aedes aegypti* from Ouagadougou, Burkina Faso

**DOI:** 10.1186/s41182-025-00888-1

**Published:** 2025-12-29

**Authors:** Hyacinthe K. Toe, Soumanaba Zongo, Florence Kabore, Inoussa Toe, Antoine Sanou, Siaka Debe, Moussa W. Guelbeogo, Moussa Namountougou, Olivier Gnankine

**Affiliations:** 1 Laboratoire de Recherche, Centre National de Recherche et de Formation sur le Paludisme, Ouagadougou, Burkina Faso; 2 Unité de Formation et de Recherche en Sciences de la vie et de la Terre, Université Joseph KI-ZERBO, Ouagadougou, Burkina Faso; 3 Unité de Formation et de Recherche en Sciences de la vie et de la Terre, Université Nazi BONI, Bobo-Dioulasso, Burkina Faso; 4 Université Yembila Abdoulaye TOGUYENI, Fada N’Gourma, Burkina Faso; 5 Centre MURAZ, Bobo-Dioulasso, Burkina Faso

**Keywords:** *Aedes aegypti*, Dengue, Insecticides resistance, Kdr mutations, Breeding containers, Ouagadougou

## Abstract

**Background:**

*Aedes aegypti*, the primary vector of Dengue fever in Burkina Faso, breeds in a variety of domestic and peri-domestic water holding containers. The influence of these water containers on the mosquitoes’ ability to survive exposure to chemical insecticides remains unclear. This study investigated the insecticide susceptibility profile of *Aedes aegypti* in relation to larval habitat types in three districts of Ouagadougou.

**Methods:**

Adult females reared from larvae collected in “domestic containers” and “car tires” were exposed separately to papers impregnated with deltamethrin, pirimiphos-methyl, and bendiocarb to determine their susceptibility profiles. A subsample of mosquitoes per locality and container type was screened for the F1534C, V1016I and V410L kdr mutations involved in pyrethroid resistance.

**Results:**

Mosquito population from the three localities showed high resistance to deltamethrin and pirimiphos-methyl and moderate resistance to bendiocarbe, with mortality rates ranging from 15% to 27%, 21% to 33% and 67% to 86%, respectively. Mosquitoes from the "domestic containers" were significantly more resistant to deltamethrin than those from tires (10% vs. 22%, *p* < 0.002). The frequency of the 1534C mutation was also significantly higher in the "domestic containers" compared to those from tires (0.88 vs. 0.76, *p* = 0.013).The other mutations 1016I and 410L, were reported with an overall frequency of 0.51 and 0.36, respectively.

**Conclusions:**

These findings suggest that larval habitat type may influence both the level and mechanisms of resistance in *Aedes aegypti*. This has important implications for the design of targeted vector control strategies in dengue-endemic settings.

**Supplementary Information:**

The online version contains supplementary material available at 10.1186/s41182-025-00888-1.

## Background

Mosquito of the *Aedes* genus, particularly *Aedes aegypti*, are primary vectors of several emerging and re-emerging arboviral diseases worldwide, including Dengue, Chikungunya, and Zika [1]. According to the World Health Organization (WHO 2022), these arboviral infections cause several million cases annually, posing an increasing threat to public health, especially in tropical regions. In West Africa, rapid and often unplanned urbanization has fostered environments highly conducive to the proliferation of *Ae. aegypti*, particularly in urban settings, where artificial larval habitats are widespread [2]. In Burkina Faso, the resurgence of vector-borne diseases is a growing concern, as evidenced by Dengue and Chikungunya outbreaks in 2023 [3] and the circulation of Zika Virus [4]. These events highlight the urgent need to strengthen surveillance and control strategies targeting *Ae. Aegypti* populations. The use of insecticide against both larvae and adult lifestages remains a widely employed method in response to outbreaks. However, the emergence of resistance in *Aedes* populations in Africa [5, 6] threatens the effectiveness of vector control interventions. The pyrethroid resistance in *Aedes aegypti* from Burkina Faso is widely spread and increasing overtime [7, 8], whereas resistance to carbamates and organophosphate remains limited [9, 10]. Previous studies assessing the susceptibility profile have relied on eggs or larval collection without considering the types of breeding sites. Resistance development may be influenced by various factors, including the nature of larval habitats, where immature stages develop [11]. The larval environment has been reported as a key factor affecting insecticide susceptibility variation in *An*. *gambiae* and *Anopheles stephensi* [12]. *Aedes aegypti* from Burkina Faso breed in diverse types of water holding containers with used tires commonly reported as the most predominant and productive larval habitats [13–15]. Physicochemical parameters of container water have also been reported to impact immature stages' productivity [16]. There are growing concerns about the sources of insecticide selection pressure in *Aedes* mosquitoes, and the type of water holding containers may play a role in influencing the insecticide susceptibility profiles. We hypothesized that domestic containers, often located near human dwellings might be exposed to household insecticides or other chemical pollutants. These micro-environments could favor the selection of resistant individuals, which may preferentially oviposit in domestic containers, unlike used tires typically found outdoors. Such differential exposure could generate distinct selection pressures depending on the breeding site type. Therefore, gaining a deeper understanding of the relationship between larval habitat types and resistance profiles is crucial for elucidating the selection patterns and guiding sustainable vector control strategies. In this study, we assessed the susceptibility profiles of *Ae*. *Aegypti* populations to common insecticide classes and examined the association between the type of breeding site and resistance level.

## Materials and methods

### Study sites

**Fig. 1:**
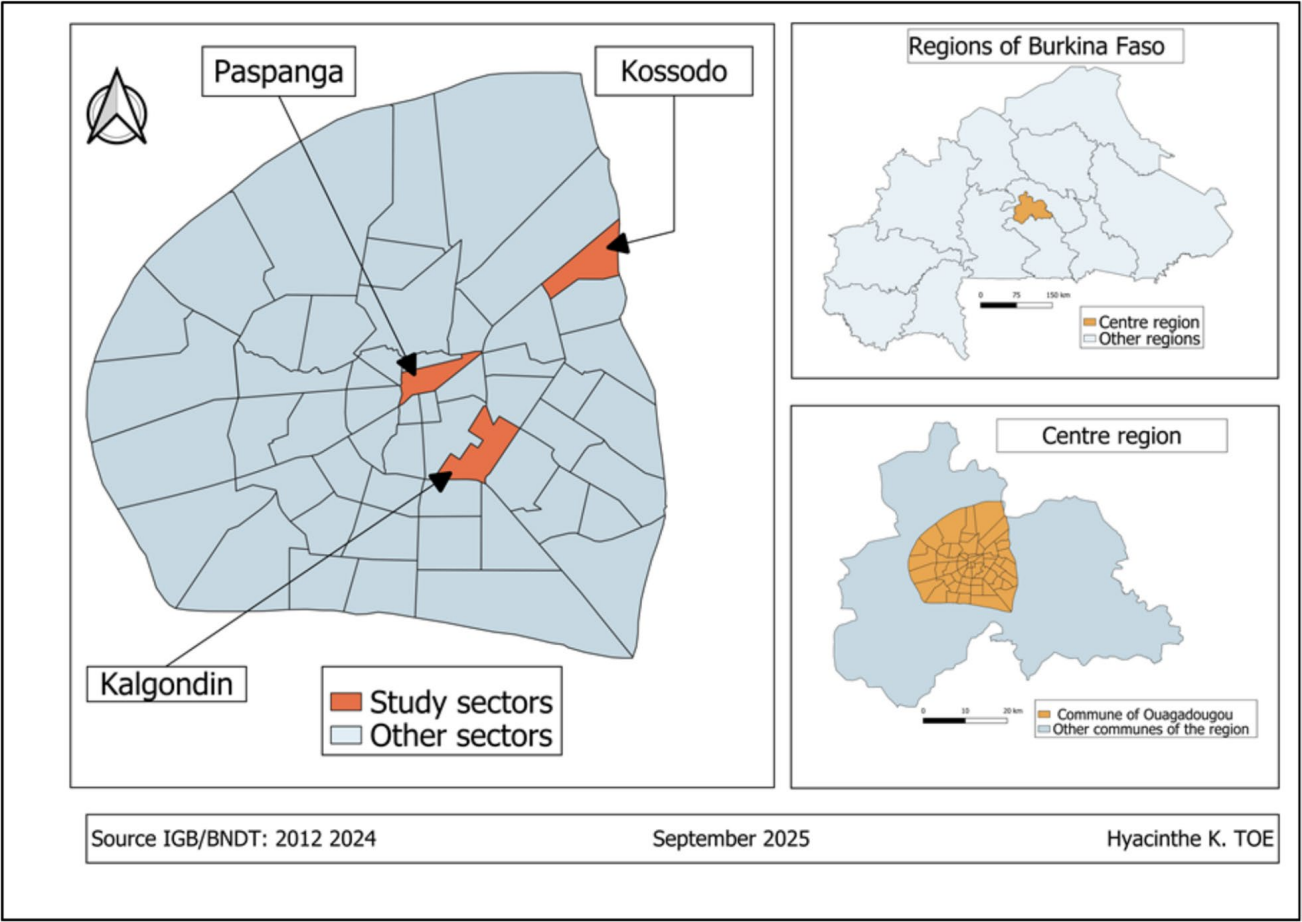
Map of Ouagadougou municipality showing the study districts

The study was conducted from mid to end of the rainy season, September to November 2021 in Ouagadougou city (12° 22ʹ N, 001° 31ʹ W), the capital and the largest city of Burkina Faso. Ouagadougou is located in the central region with an arid savannah (soudano-sahalian) climate, annual rainfall of 780 mm, and mean temperature of 28 ℃ [17]. The city is divided into twelve boroughs and sampling was performed in three of them: Kalgondin (12° 20′ 40.15′′ N, 1° 30′ 5.42′′ W), is adistrict from sector 24 in the borough N°5. Paspanga (12° 22′ 50.65′′ N, 1° 30′ 34.54′′ W), an area from sector 10 within borough N°2 and Kossodo (12° 25′ 9.57′′ N, 1° 27′ 59.48′′ W) located in sector 15 within borough N° 4 (Fig. 1).

### Larvae sampling and processing in the laboratory

**Fig. 2:**
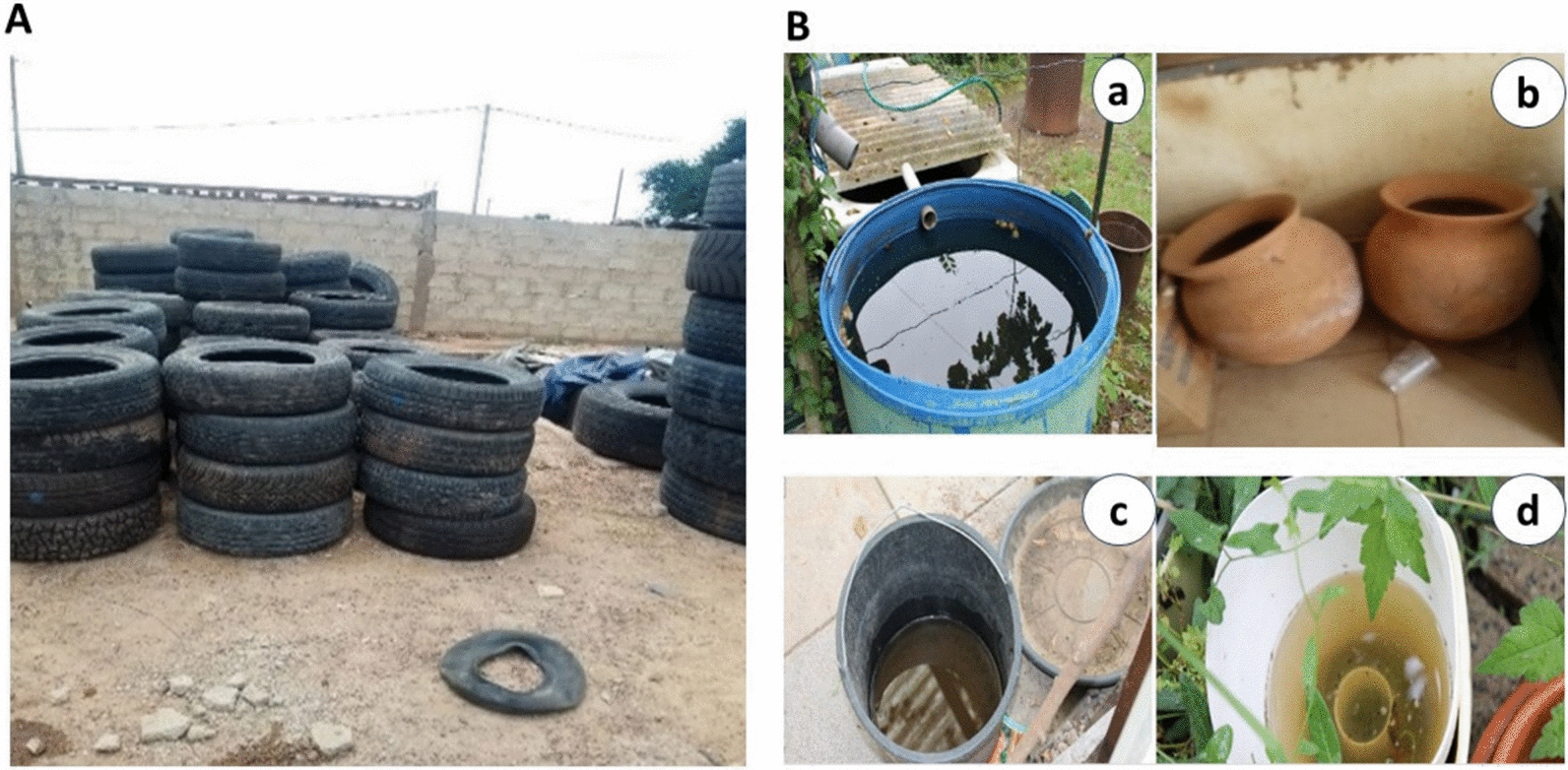
Classified containers type, **A** car tires, **B** domestic containers (**a** drum/barrel, **b** terracotta jars, **c** buckets, and **d** animal feeding pots)

*Aedes* larvae were collected in diverse types of water holding containers, commonly found across the three districts of Ouagadougou. Collection were conducted over several days (at least 5 days per site) to obtain a sufficiently large, diverse and mixed sample of larvae. The water-holding containers were categorized into two groups: car tires and domestic containers (DCs). The DCs included household’s items such as drums/barrels, buckets, terracotta jars, animal feeding pots, etc. (Fig. 2A) found into the households. In contract, car tires (Fig. 2B) were found mostly outside human dwellings. Overall, a total of 819 domestic containers and 1106 car tires were sampling. Specifically, 210, 509 and 100 DCs were sampled in Bogodogo, Kossodo and Paspanga, respectively, while 306, 600 and 200 were sampled in these same districts.

Larvae collected in the field were transferred separately in 1.5L bottles and pooled according to the category of water holding containers once at the insectary. Larvae were reared to adulthood under controlled insectary conditions of temperature (27 ± 2 °C) and relative humidity (75 ± 10%RH). Pupae were put separately in the cage according to the type of water containers for emergence and adults were supplied with a 10% glucose solution.

### WHO susceptibility bioassays

For each bioassay, one hundred mosquitoes (four replicates of 25 mosquitoes) of 3–5 day non-blood fed females from Kalgondin, Paspanga and Kossodo were exposed to 0.05% deltamethrin, 0.1% bendiocarb and 0.21% pirimiphos-methyl following the WHO tube bioassay procedure [18]. The susceptibility profile was determined per insecticide and container type based on the mortality rate recorded 24 h after exposure, and classified as resistant, susceptible, or probable resistance following WHO criteria [18]. The *Ae*. *aegypti* Bora Bora populations were tested on all insecticides impregnated papers as a control to ensure their quality. Control mosquitoes (*N* = 20–25 females per test population and the Bora Bora strain) were exposed for the same duration to untreated filter papers. Control, dead, and surviving mosquitoes after each test were stored with silica gel in 15 ml tubes for subsequent kdr mutations genotyping.

### Investigation of kdr mutations resistance mechanisms

A subset of 186 unexposed *Ae. aegypti* mosquitoes from the three districts’ sites were screened to determine the frequency of the F1534C, V1016I, and V410L kdr mutations. Mosquitoes from tires and domestic materials were analyzed separately. Genomic DNA was extracted from whole mosquitoes using genomic DNA isolation reagent from DNAzol® Reagent (Invitrogen by Thermo Fisher Scientific) according to the manufacturer’s guidelines. Real-time melting curve qPCR analyses were performed to genotype the F1534C, V1016I and V410L *Aedes* kdr mutations using specific primers and PCR conditions (Table S1), as described by to Saavedra-Rodriguez et al., [19, 20].

The frequency of kdr mutations were determined empirically/manually as follows: *f*(*R*) = [2 × *n*(RR) + *n*(RS)]/(2 × *N*), where *n*(RR) and *n*(RS) represent the number of homozygous and heterozygous individuals carrying the resistant allele, respectively, and *N* is the total number of individuals genotyped (i.e., 2*N* alleles observed).

### Statistics analysis

The 24 h post-exposure mortality rates were calculated per insecticide and per type of breeding site. The *Aedes* mosquito population was classified as resistant when mortality was less than 90% and susceptible for mortality higher than 98%. However, resistance was suspected when the mortality rate was between 90% and 98% [18]. The mortality rate for each insecticide was compared between collection sites and between breeding sites. A Generalized Linear Model (GLM) was separately performed for each insecticide to assess variability in the mortality according to the sites and containers types using R software. The allelic frequency of kdr mutations were determined empirically/manually as follows: (*R*) = [*2* × *n*(RR) + *n*(RS)]/(2 × *N*), where *n*(RR) and *n*(RS) represent the number of homozygous and heterozygous individuals carrying the resistant allele, respectively, and *N* is the total number of individuals genotyped (i.e., 2*N* alleles observed). The comparison of the allelic frequency between sites and breeding sites was performed using the chi-square test calculator from the DATA tab [21].

### Ethical considerations

This study was approved by the Ethics Committee on the date of deliberation of 03 Sept 2019 under Number N°2020/0001/MS/SG/INSP/CNRFP/CIB; with the theme of protocol: Evidence of Community Engagement and Participation in Dengue Vector Control in Urban Areas of Burkina Faso.

## Results

A total of 1,802 adult *Ae*. *Aegypti* mosquitoes, reared from larvae collected in the three district sites, were tested for susceptibility to deltamethrin 0.05% (pyrethroids), bendiocarb 0.1% (carbamates), and pirimiphos-methyl 0.21% (organophosphates), following WHO guidelines [18] (Table S2).

### *Aedes aegypti* overall susceptibility profile to insecticides

**Fig. 3:**
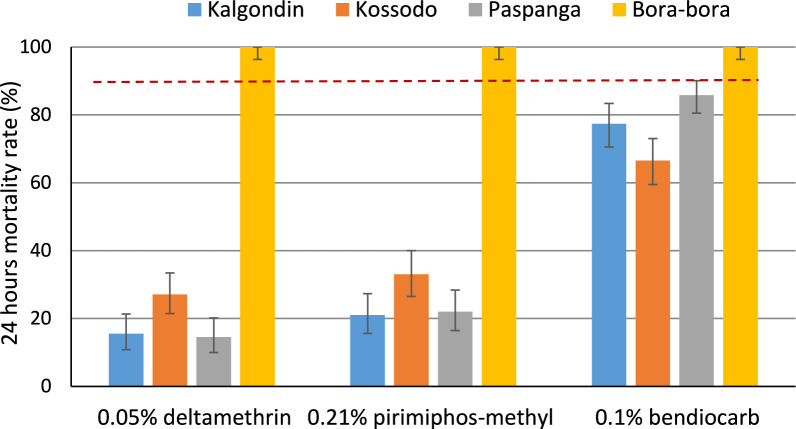
Overall insecticide susceptible profile to 0.05% deltamethrin, 0.1% bendiocarb and 0.21% pirimiphos-methyl in *Aedes aegypti* from the three districts of Ouagadougou, (September 2022). The dotted line indicates the threshold of 90%. Error bars indicate 95% binomial confidence intervals (CIs) for the considered insecticide and locality

*Aedes aegypti* populations from the three districts exhibited high levels of resistance to deltamethrin, with mortality rates below 30%. A similarly strong resistance was observed against pirimiphos-methyl, with mortality rates below 40%. Only bendiocarb demonstrated relatively moderate effectiveness, with mortality rates above 56%, reaching up to 91.2% in Paspanga (Fig. 3).

### Variability of insecticide susceptibility profile according to the type of breeding sites

#### Susceptibility of *Aedes aegypti* to pyrethroids

**Fig. 4:**
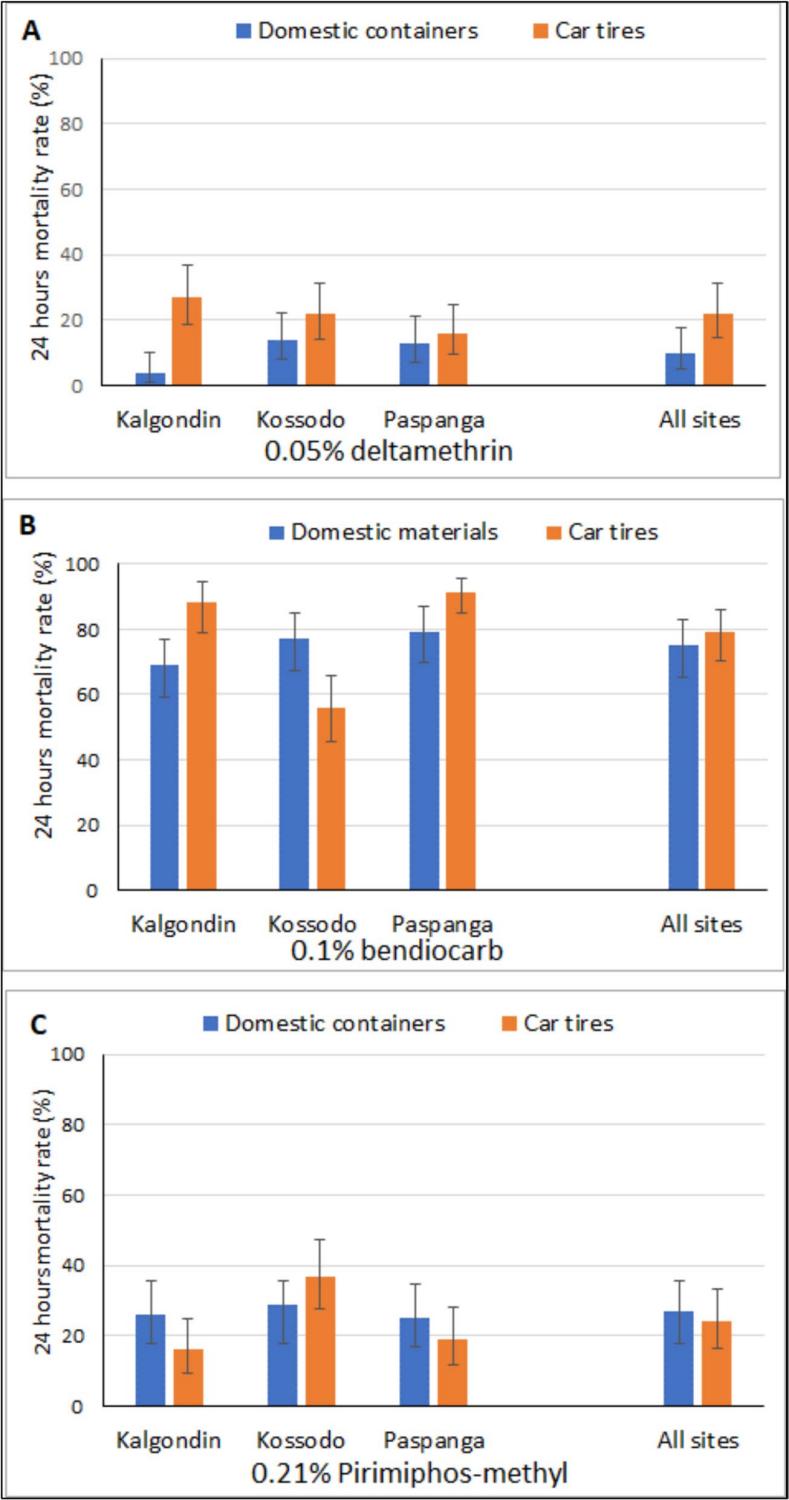
Mortality rates to 0.05% deltamethrin (**A**), 0.1% bendiocarb (**B**) and to 0.21% pirimiphos-methyl (**C**) according to the breeding types in *Aedes aegypti* from the district of Kalgondin, Kossodo and Paspanga. Error bars indicate 95% binomial confidence intervals (CIs) for the considered insecticide, breeding site type and locality

Mortality rates observed after 24 h of exposure to 0.05% deltamethrin ranged from 4% to 27% across the study sites (Table S1 and Fig. 3). All values were well below the 90% threshold set by WHO for susceptibility, indicating confirmed resistance in all localities. Overall, there was no significant variation in the mortality rates between sites when combining all the container types (*p* = 0.6195). However, when considering the breeding site type, the mortality rate was found significantly lower in domestic containers than those from car tires (*p* < 0.001), with an overall mortality rate of 10% and 22%, respectively. The lowest mortality rates were recorded among *Aedes aegypti* populations originating from domestic containers, with rates of 4%, 13%, and 14% in Kalgondin, Paspanga, and Kossodo, respectively (Fig. 4A). In contrast, populations collected from tires exhibited mortality rates of 27%, 16%, and 22% in the same sites.

### Susceptibility of *Aedes aegypti* to carbamates

Mortality rates following 24-h exposure to bendiocarb 0.1% ranged from 76% to 86% showing a significant variation according to the district (*p* < 0.0001). All localities exhibited mortality rates below 90%, indicating a resistant population according to WHO standards. (Table S2 and Fig. 3).When comparing mortality rates between types of breeding sites, no statistical difference was observed (*p* = 0.2674). The overall mortality rates were 75% in domestic containers vs. 79% in car tires (Fig. 4B).

### Susceptibility of *Aedes aegypti* to organophosphates

Mortality rates observed after 24 h of exposure to pirimiphos-methyl varied significantly among sites (*p* = 0.0103), ranging from 16% to 37% (Table S2 and Fig. 4C). However, no significant difference was observed between container types (*p* = 0.4526), with overall mean mortality rates of 27% for domestic containers and 24%for car tires (Fig. 4C).

### Genotyping of F1534C, V1016I, and V410L mutations in *Aedes aegypti*

A total of 186 *Ae. aegypti* specimens were successfully genotyped for the F1534C, V1016I, and V410L mutations (Table 1). The F1534C mutation was reported with an overall frequency of 0.82. The frequency was found to vary significantly according to the districts (*p* < 0.0001). The lower frequency was recorded in Kalgondin district (0.67, 95% CI 0.58–0.75), while it was nearly fixed in Paspanga, reaching 0.98 (95% CI 0.96–1.00).

**Table 1:** Frequency of F1534C, V1016I and V410L in unexposed *Ae. aegypti* from the three study sites in the city of Ouagadougou

	F1534C mutation					V1016I mutation				V410L mutation			
Locality	Total	Genotype	*n*	*f*(1534C)	*p* value	Genotype	*n*	*f*(1016I)	*p* value	Genotype	*n*	f(410L)	*p* value
Bogodogo	63	RR	37	0.67	< 0.0001	RR	6	0.42	0.057	RS	16	0.32	0.12
		RS	10			RS	41			RS	8		
		SS	16			SS	16			SS	39		
Kossodo	64	RR	40	0.81		RR	13	0.55		RS	13	0.33	
		RS	24			RS	45			RS	16		
		SS	0			SS	6			SS	35		
Paspanga	59	RR	57	0.98		RR	8	0.55		RS	4	0.43	
		RS	2			RS	49			RS	43		
		SS	0			SS	2			SS	12		
Total	186	RR	134	0.82		RR	27	0.51		RS	33	0.36	
		RS	36			RS	135			RS	67		
		SS	16			SS	24			SS	86		

**RR**: homozygote for resistant allele, **SS**: homozygote for wild type allele, **RS**: heterozygotes

3-2-1 Kdr target site mutations and their variability according to the type of breeding sites

The V1016I ranged from 0.42(95% CI 0.47–0.64) in Kalgondin to 0.55 in Kossodo and Paspanga, with an average of 0.51. The frequency was not found significantly different between districts (*p* = 0.057). Regarding the V410L mutation, its frequency ranged from 0.32 [95% CI 0.24–0.40] in Kalgondin, 0.33 [95% CI 0.25–0.41] in Kossodo, and to 0.43 (95% CI 0.34–0.52) in Paspanga with an average of 0.36. The frequency was not varied according to the districts. The F1534C mutation was by far the most prevalent (0.82, 95% CI 0.78–0.86) among the three kdr mutations screened. A statistical comparison revealed a significant difference in the frequency between the three mutations (*p* < 0.0001), Table 2.

**Table 2:** Frequencies of F1534C, V1016I and V410L mutations in unexposed *Ae. aegypti* from tires and domestic containers across the three study sites of Ouagadougou

Type of breeding sites	F1534C mutation				V1016I mutation				V410L mutation			
	Genotype	*n*	*f*(1534C)	*p* value	Genotype	*n*	*f*(1016I)	*p* value	Genotype	*n*	*f*(410L)	*p* value
Car tires	RR	63	0.76	0.013*	RR	15	0.56	0.75	RR	22	0.34	0.82
	RS	16			RS	74			RS	19		
	SS	14			SS	4			SS	52		
Domestic containers	RR	71	0.88		RR	19	0.54		RR	10	0.33	
	RS	19			RS	61			RS	40		
	SS	2			SS	12			SS	42		

*Significant *p* value (*p* < 0.05)

**RR**: homozygote for resistant allele, **SS**: homozygote for wild type allele, **RS**: heterozygotes

The genotyping results were analyzed by type of breeding site (Table 2).The frequency of F1534C mutation showed a significant difference between types of breeding sites (*p* = 0.013), with a higher frequency recorded in domestic containers (0.88) compared to car tires (0.76). In contrast, the V1016I and V410L mutation exhibited nearly identical frequencies for both types of breeding sites. The frequency of V410L was 0.34 in car tires vs. 0.33 in domestic containers, while the frequency of V1016I was 0.56 and 0.54, respectively (in bold a significant p-value, < 0.05).

## Discussion

Effective control of arboviral diseases depends on a thorough understanding of the bio-ecology of their mosquito vectors. This includes identifying key larval habitats and managing insecticide resistance, particularly in the context of increasing reports of widespread resistance involving multiple mechanisms in *Aedes* populations worldwide [22].

This study assessed the susceptibility profile of *Ae*. *aegypti* in relation to breeding sites across three districts of Ouagadougou, Burkina Faso. The results reported multiple resistances to pyrethroids, carbamates and organophosphates with particular strong resistance observed to deltamethrin and pirimiphos-methyl. Resistance of *Aedes* mosquitoes to the main insecticide classes is now well-documented in Africa [5, 6]. Pyrethroid resistance, in particularly, has been widely reported in Burkina Faso [7, 8, 10] and throughout Sub-Saharan Africa [23–26]. Resistance to carbamates and organophosphates although less common, has also been reported [9]. Multiple resistance to pyrethroids, carbamates and organophosphates is relatively uncommon in *Ae. aegypti*, but it has already been reported in Burkina Faso, particularly in urban environments. High levels of resistance are particularly observed in urban settings and may be linked to both genetic mutations and changes in metabolic enzyme activity. Previous studies in Burkina Faso have demonstrated the presence of detoxification enzymes associated with pyrethroid resistance in *Ae*. *aegypti* from Bobo-Dioulasso and Ouagadougou [10]. The widespread use of insecticides, combined with kdr flow and the urban adaptation of *Ae*. *aegypti*, may increase mosquito exposure to insecticides and consequently intensify the selection pressure for resistance. Similarly, in *An gambiae sl*, for instance, the selection of resistance has been strongly linked to both the public health and agricultural use of insecticides, which can contaminate mosquito breeding sites [27–31].

High frequencies of the F1534C mutation and moderate frequencies of V1016I and V410L kdr mutations were reported in the three districts. The frequencies of these mutations did not vary significant across districts, suggesting a genetically homogeneous of *Ae. aegypti* population within Ouagadougou and possible local gene flow. Indeed, studies from Mexico have shown that substantial migration and gene flow can occur among *Aedes* populations up to 150 km apart [32–34]. The three kdr mutations showed different levels of frequencies. The 1534C allele was found at high frequency and nearly fixed in Ouagadougou, whereas 1016I and V410L occurred at moderate frequencies that appear to be increasing over time [7]. These alleles have been consistency associated with deltamethrin and permethrin resistance in *Ae. aegypti* from Ouagadougou. The F1534C mutation, for instance has been linked to survival after 4 h of exposure to deltamethrin [9], while the combined 1534C/1016I/410L haplotype has been associated with permethrin resistance [35].The widespread occurrence of pyrethroid resistance in *Aedes* populations calls for an urgent exploration of alternative control strategies, including plant-derived products [36–38].

A particularly interesting finding from this study is the potential influence of breeding conditions, especially the type of water holding container, on pyrethroid resistance levels. A significantly high frequency of the 1534C was recorded in populations emerging from domestic containers, previously with high deltamethrin resistance [9]. This raises questions about the role breeding container type might play in resistance selection in *Aedes* mosquitoes. Does selection occur primarily during the adult life stage, before females oviposit in these containers? Adult Aedes often rest near their breeding sites. Those emerging from domestic containers are likely to remain close to human dwellings, where they may be exposed to household insecticides. In contrast, adults emerging from tires, which are often located or in peri-domestic areas, may experience less exposure to domestic insecticides. Selection could also occur during larval stage. How could the type of breeding containers affect the insecticide resistance? Exposure of larvae to sub lethal doses of xenobiotics or insecticides has been shown to increase their tolerance to insecticides and induce over expression of resistance-related genes[39].

In *Anopheles gambiae* sl, the selection of insecticide resistance during the larval stage has been primarily linked to the presence of agrochemical or pesticide residues in breeding waters [27, 29, 40]. This study, however, did not assess the presence of xenobiotic or chemical pollutants in the larval habitats. Future research should include such analysis and also investigate other resistance mechanisms, such as metabolic-based mechanisms, which may offer additional understanding of the higher 1534C allele and deltamethrin resistance observed in *Aedes* from domestic containers, as the link between kdr-based mutations and phenotypic resistance can sometimes be inconsistent [41].

Although these findings enhance our understanding on the influence of *Aedes aegypti* breeding sites on insecticide resistance, the study has some limitations. Further investigations conducted over a longer sampling period, with an increased number of replicates in different settings, such as varying levels of urbanicity, are needed. In addition, obtaining information on environmental chemical composition as well as the biotic and abiotic parameters associated with breeding site water, would broaden our understanding of how *Aedes aegypti* breeding sites and their surrounding environment contribute to the development of insecticide resistance.

## Conclusion

The study documents the presence of multi-class insecticide resistance in *Aedes aegypti* populations from Ouagadougou, involving pyrethroids, carbamates and organophosphate insecticide classes. Significantly high deltamethrin resistance was observed in mosquitoes derived from domestic containers compared to those from car tires. This increased resistance is supported by the elevated frequency of resistance-associated kdr mutations. Future research should broaden larval sampling and investigate the physicochemical parameters of breeding sites water, as well as environmental contaminants to elucidate habitat-specific resistance mechanisms.

## Supplementary Information

Additional file1

Additional file2

## Data Availability

The data sets generated during and/or analyzed during the current study are available from the corresponding author on reasonable request.
