# Supplementary material for: Multiple resistance and influence of breeding sites on pyrethroid resistance in Aedes aegypti from Ouagadougou, Burkina Faso
Source: Trop Med Health. 2025 Dec 29;53:194. doi: 10.1186/s41182-025-00888-1 (PMC12746623; doi:10.1186/s41182-025-00888-1)
Supplement: Supplementary file 1 — Additional file1 [file 41182_2025_888_MOESM1_ESM.docx]

**Table S1:** Primers list, their sequences and qPCR program used for detecting F1534C, V1016I, V410L kdr mutations using the melting curve analysis method
