# Supplementary material for: Multiple resistance and influence of breeding sites on pyrethroid resistance in Aedes aegypti from Ouagadougou, Burkina Faso
Source: Trop Med Health. 2025 Dec 29;53:194. doi: 10.1186/s41182-025-00888-1 (PMC12746623; doi:10.1186/s41182-025-00888-1)
Supplement: Supplementary file 2 — Additional file2 [file 41182_2025_888_MOESM2_ESM.docx]

**Table S2**: Raw data of WHO susceptibility tube tests with total number of *Aedes aegypti* mosquito tested, total dead and the mortality rates following exposure to 0.05% deltamethrin, 0.1% bendiocarb, and 0.21% pirimiphos-methyl. The data are presented per locality and containers type.
